# Severe Basilar impression in osteogenesis imperfecta treated with halo gravity traction, occipitocervicothoracic fusion, foramen magnum and upper cervical decompression and expansive duroplasty: a technical note

**DOI:** 10.1007/s00381-022-05495-7

**Published:** 2022-03-16

**Authors:** Gianpaolo Jannelli, Alessandro Moiraghi, Luca Paun, Enrico Tessitore, Romain Dayer, Andrea Bartoli

**Affiliations:** 1 Department of Neurosurgery, Faculty of Medicine, Geneva University Hospitals and University of Geneva, Geneva, Switzerland; 2 Department of Neurosurgery, GHU Paris - Sainte-Anne Hospital, Paris, France; 3 Université de Paris, Sorbonne Paris Cité, Paris, France; 4 IMA-Brain, Institut de Psychiatrie Et Neurosciences de Paris, Inserm, U1266 Paris, France; 5 Departments of Pediatric Orthopedics, Geneva University Hospitals, Geneva, Switzerland

**Keywords:** Basilar impression, Osteogenesis imperfecta, Brainstem compression, Chiari malformation, Hydrocephalus, Halo traction

## Abstract

Osteogenesis imperfecta (OI) is a rare bone disease due to an abnormal synthesis of 1-type collagen. OI is frequently associated with basilar impression (BI), defined by the elevation of the clivus and floor of the posterior fossa with subsequent migration of the upper cervical spine and the odontoid peg into the base of the skull. Bone intrinsic fragility leading to fractures and deformity, brainstem compression and impaired CSF circulation at cranio-vertebral junction (CVJ) makes the management of these conditions particularly challenging. Different surgical strategies, including posterior fossa decompression with or without instrumentation, transoral or endonasal decompression with posterior occipito-cervical fusion, or halo gravity traction with posterior instrumentation have been reported, but evidence about best modalities treatment is still debated. In this technical note, we present a case of a 16-years-old patient, diagnosed with OI and BI, treated with halo traction, occipito-cervico-thoracic fixation, foramen magnum and upper cervical decompression, and expansive duroplasty. We focus on technical aspects, preoperative work up and postoperative follow up. We also discuss advantages and limitations of this strategy compared to other surgical techniques.

## Introduction

OI is a rare bone disease due to an abnormal synthesis of 1-type collagen. OI is frequently associated with several conditions, like fractures, kyphoscoliosis or CVJ anomalies [1]. These patients are often diagnosed (25–71%) with BI, defined by the elevation of the clivus and floor of the posterior fossa with subsequent migration of the upper cervical spine and the odontoid peg into the base of the skull [2–8]. This condition can be asymptomatic or present with different neurological complications. Indeed, progressive brainstem compression can lead to neurological deficit and impaired CSF circulation with symptomatic hydrocephalus and secondary Chiari I malformation.

Treatment options include posterior fossa decompression with or without instrumentation, transoral or endonasal decompression with posterior occipito-cervical fusion, or halo traction with posterior instrumentation [1, 4, 6, 9–11]. However, considering the rare incidence of this condition, evidence about best modalities treatment is scarce [4].

We describe a case of OI and BI, treated with Halo traction, occipito-cervico-thoracic fusion, foramen magnum and upper cervical decompression and expansive duroplasty. We will focus on technical aspects as well as on advantages and limitations of this strategy.

## Case description

**Fig. 1:**
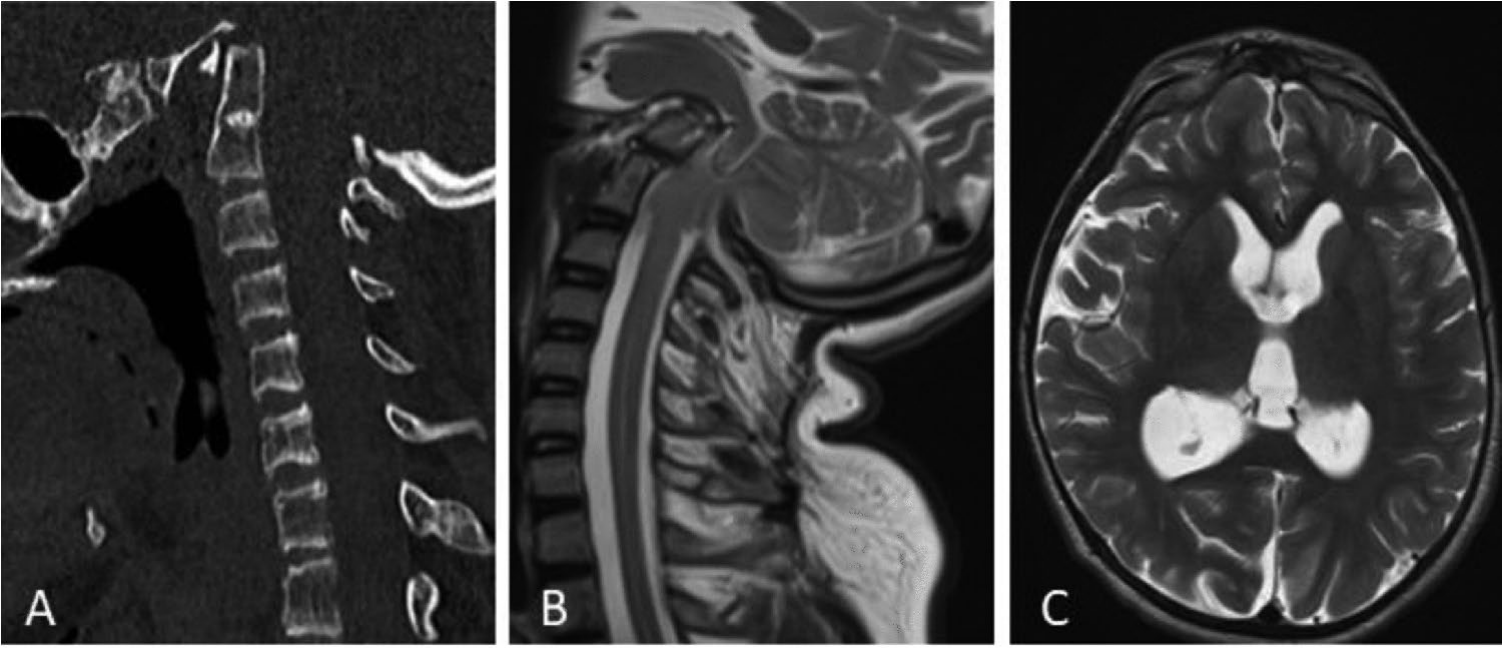
Preoperative imaging**: A** Sagittal CT scan showing basilar impression. **B** Sagittal T2-weighted MRI showing brainstem compression due to prolapse of the dens in the foramen magnum. **C** Axial T2-weighted MRI showing hydrocephalus due to CSF impaired circulation at the CVJ

**Fig. 2:**
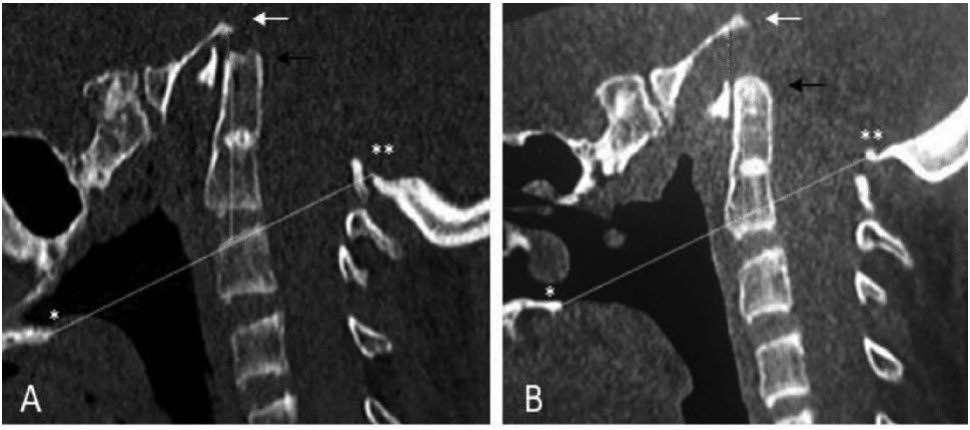
Pre-and postoperative BI measures on sagittal CT scan: the Chamberlain’s line (yellow line) is drawn from the posterior surface of the hard palate (*) to the tip of the Opisthion (**). The basilar-dens interval (red line), measured from the Basion (white arrow) to the tip of the dens (black arrow). **A** CT scan of CVJ before Halo-traction: the tip of the dens (black arrow) is placed 30 mm above this line while the basilar-dens interval was 3 mm. **B** CT scan of CVJ after 6 weeks of treatment by Halo-traction: the tip of the dens was placed 20 mm above the Chamberlain line, with a basilar-dens interval passing from 3 to 9 mm

A 16-years-old boy was known for type III OI, BI, mesenchymal scoliosis and a previous history of long bone fractures. BI was clinically silent until the age of 15, when he presented with symptoms of raised intracranial pressure, central apnea, impaired cervical motion and progressive gait disturbances. Cervical CT showed severe deformity involving thoracic, cervical spine and the CVJ, with the tip of the dens placed 30 mm above the Chamberlain line and a basion-dens interval of 3 mm. Cerebral MRI found brainstem compression associated with Chiari I malformation and signs of hydrocephalus (Fig. 1). Polysomnography did show severe obstructive sleep apnoea with no clear correlation with the positioning of the patient during sleep. There were as well central apnoea components with moderate nocturnal hypoxemia and alveolar hypoventilation. We decided to treat first the deformity by a Halo traction, with a significant improvement at 6 weeks radiological follow-up (a second polysomnography performed after halo positioning detected no significant change over the period of traction) (Fig. 2). The second stage involved an occipito-cervico-thoracic fusion, foramen magnum decompression with upper cervical laminectomy and expansive duroplasty.

## Surgical procedure

**Fig. 3:**
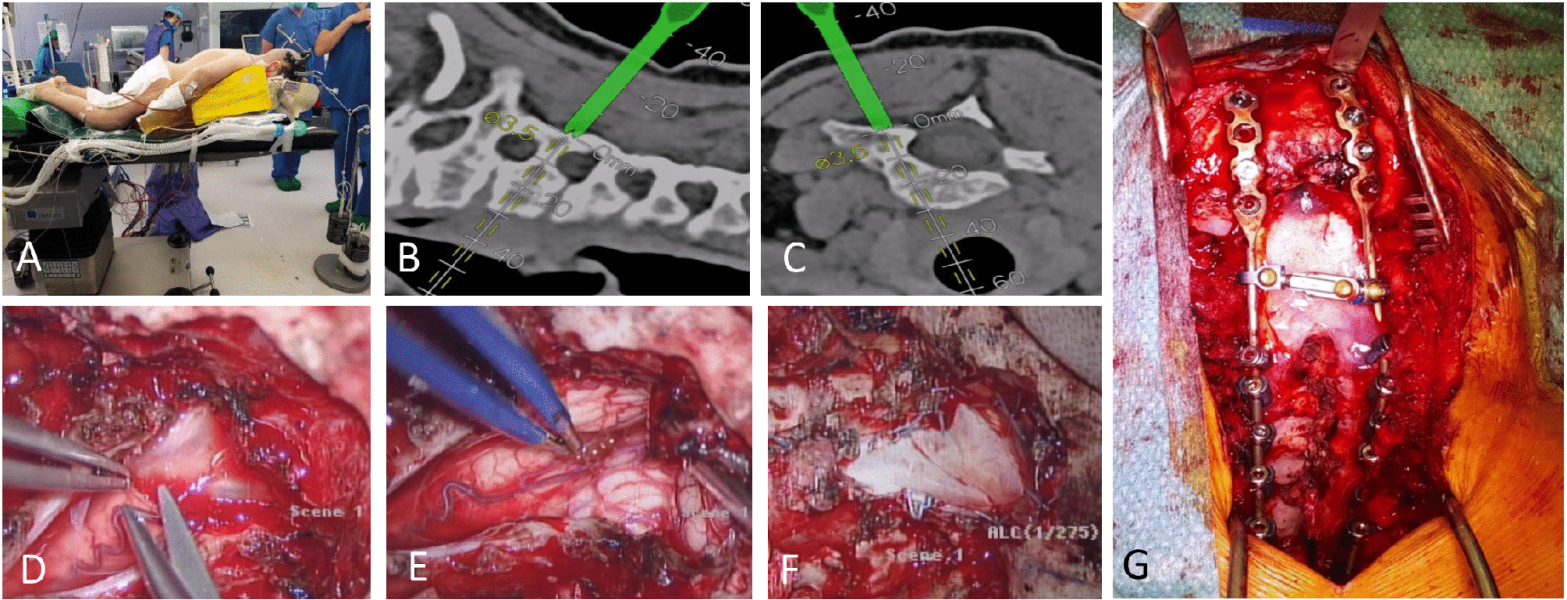
Intraoperative setup and images. **A** Surgical positioning: patient’s neck is hyperextended after the patient is fully positioned in prone position, permitting to straighten up the cervical spine. Halo-traction remain attached during the surgery to help maintaining deformity correction whilst the patient is monitored with sensory-motor evoked potentials. **B, C** Pedicle screws trajectory verified with intraoperative navigation system. **D** Durotomy is performed with a Y-cut permitting a larger exposure of the cisterna magna. IV ventricle is opened throughout Magendie foramen. **E** A wide decompression of the brainstem, cerebellar tonsils and first cervical segments is achieved. **F** A Xenosure Biologic Patch, Duraseal and Tachosil are used for a watertight duroplasty. **G** A C0-C4 cross-link connector strengthens the fixation

The patient lied in prone position on a radiolucent operative table. The head was flexed in the Halo traction (6 kg) to maintain the deformity correction obtained preoperatively (Fig. 3a). The rest of intraoperative set-up consisted of 3D C-arm fluoroscopy, spinal navigation, intraoperative ultrasound, surgical microscope and neuromonitoring (XI and XII cranial nerve and sensory-motor evoked potentials, which did not show any change during positioning and the whole intervention).

The skin was incised from the external occipital protuberance to Th4. Anatomic landmarks were exposed and identified: occipital squama, foramen magnum, and posterior arches from C1 to Th2, including the entry-points on lateral masses (from C4 to C6) and pedicles (from C7 to Th2). Surface matching was performed on Th2 and C6 posterior elements using the uploaded preoperative CT. The accuracy was verified by intraoperative control of the anatomical landmarks. Under navigation guidance, C4-C6 lateral mass screws (only on the right side for C6 due to its extremely small and dysplastic left lateral mass and pedicle) and C7-Th2 bilateral pedicle screws were inserted (Fig. 3b, c). A second scan with the 3D C-arm confirmed the correct screw position.

Suboccipital craniectomy was performed taking care to preserve dura mater integrity to perform an expansive duroplasty at the end of the procedure. Then, a C1-C2-C3 laminectomy was performed.

Under microscope, the dura was opened in a “Y” fashion. Cisterna magna arachnoid was opened with an immediate CSF release (Fig. 3d). Decompression was achieved by identification of the vallecula and cerebellar tonsils, the bulbo-medullary junction and the first cervical segments of the cord. The IV ventricle was opened through the foramen of Magendie ensuring no further thick arachnoid bridge was present. (Fig. 3e).

An expansive watertight duroplasty was performed using a XenoSure Biologic Surgical Patch (Fig. 3f) and strengthened with Duraseal to reduce the risk of CSF leak.

Occipital plate was fixed inferiorly to the external occipital protuberance and above the midline craniectomy. Polyaxial screws were fixed to the rods and bolts were tightened. To minimize screw pull-out, two 3.5 titanium alloy rods were contoured, and no reduction was done before setscrews tightening. A cross-link connector was placed between C0 and C4 (Fig. 3g). The wound was standardly closed layer by layer.

The surgery was uneventful and took 9 h. The patient was postoperatively monitored in the intensive care unit.

## Postoperative follow-up

Postoperatively, difficult airways management due to a weak swallowing reflex and the degree of head flexion required temporary tracheostomy which was removed 3 months later. No new neurological deficit was observed.

**Fig. 4:**
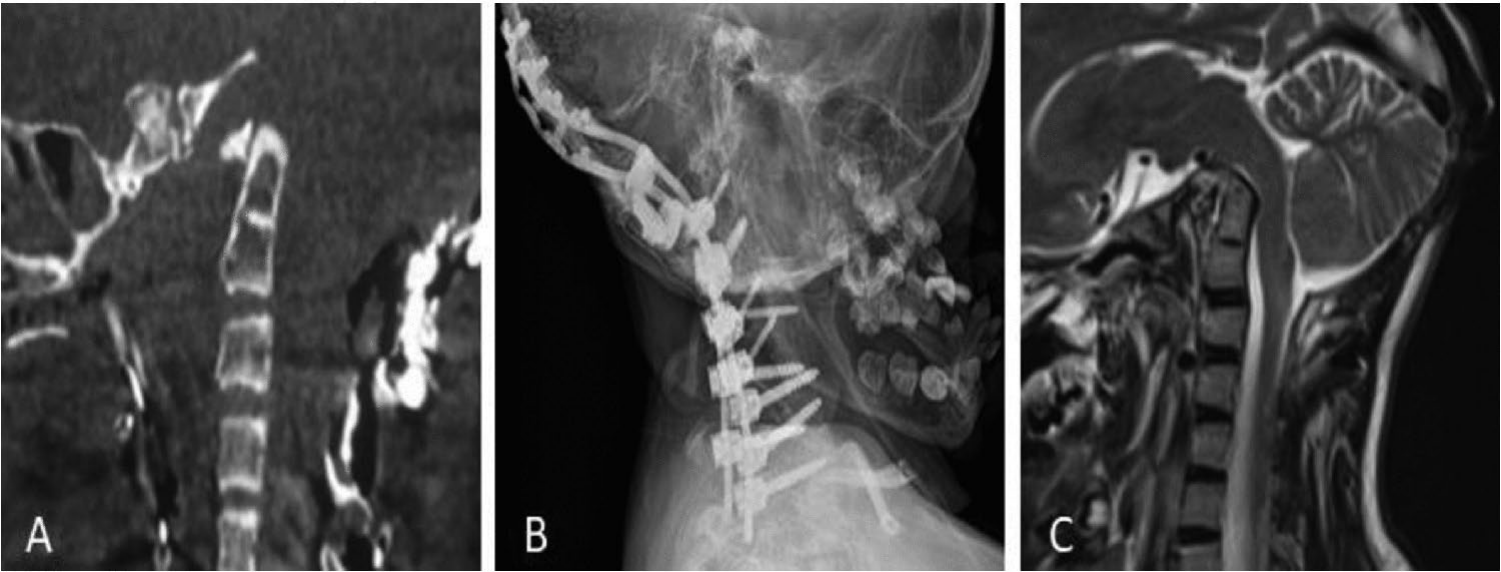
Postoperative and follow-up images. **A** Immediate postoperative CT scan showing that the deformity correction obtained with the Halo is preserved. **B** Cervical X-rays at 6 months follow-up, showing a stable reduction of the deformity. **C** MRI at 6 months follow-up, showing the posterior fossa decompression resulting in a reduced mass effect on the brainstem and restored CSF circulation at the CVJ

The correction of deformity achieved with the Halo was maintained, as confirmed at 24 h CT (Fig. 4a).

Postoperative recovery was complicated by ventriculitis observed one month after the operation, requiring an external ventricular drainage and antibiotics and subsequently a ventriculoperitoneal shunt.

At 6 months follow-up, images showed a clear reduction of BI resulting in good brainstem decompression and restoration of CSF circulation at the CVJ (Fig. 4b, c). No screw loosening was observed.

At 1-year follow-up, the patients resolved central apnea and headache. He is currently scheduled for a posterior spinal instrumented fusion from Th2 to pelvis due to progression of scoliosis. Off note, the two 4.5 rods were left long distally, to allow for easy connection in case of a long spine fusion.

## Discussion

Management of this rare association remains controversial, and different techniques have been described from the past. Suboccipital decompression with upper cervical laminectomy has been reported [4, 10], but this strategy would have hastened the progression of the ventral brainstem compression as well as of the deformity [7]. Goel et al. distinguished two types of BI, based on the absence (group I) or presence (group II) of Chiari I malformation [2]. The double posterior fossa mass effect, by the odontoid anteriorly and by the posterior rim of the foramen magnum posteriorly, suggests proceeding with transoral surgery in group I patient while decompression of the foramen magnum is suitable in Group II. In our case, anterior approaches (may it be transoral or endonasal) were not considered due the severity of the compression and the deformity and the higher risk of CSF leak and neurovascular complications [12]. Noske et al. reported a case treated initially by halo traction and posterior fusion, requiring then a shunt due persistent hydrocephalus [6]. In our case, Halo traction was managed according to specific considerations for OI patients: Six skull pins are usually inserted to try to prevent the halo pull-out that may occur with a 4 pins construct. The traction weight was gradually increased after halo application up to 6 to 8 kg (i.e. the weight of the head) for a total of 6 weeks. Therefore, the amount of weight used in halo traction for BI differs significantly from that used to treat severe scoliosis (50% of body weight).

Despite the significant range of complications associated with the technique described (tracheostomy and difficult airways management, potential screw loosening, posterior fossa hematoma, CSF leak and infection), some considerations suggested to proceed with this approach:

1. The improvement obtained with Halo traction indicated the reducible character of the BI and needed to be stabilized with a posterior instrumentation.
2. Posterior fossa decompression was necessary to achieve good brainstem decompression and restore CSF circulation.
3. In a subset of patients, the longstanding hydrocephalus can be “compensated” as reported by Noske et al. [6] and potentially not requiring a shunt.

In our opinion a sufficient decompression by suboccipital craniectomy and C1-C3 laminectomy helped to restore a normal CSF circulation, and to treat the induced Chiari I – like symptoms. On the other hand, the ventriculitis may have well decompensated the preexisting hydrocephalus requiring finally a shunt.

Moreover, we decided to extend the posterior instrumentation from the occiput to Th2. This choice was supported by some anatomical considerations which made impossible to insert screws in C1 (too high and hidden by the occipital bone so that its entry points were out of reach), C2 and C3 (C2 entry points too far and deep from the rods, extremely small C3 lateral mass). Furthermore, including the cervico-thoracic junction with bilateral pedicular screws (C7, Th1 and Th2) allowed to have a much better grip, a more solid construct and therefore reduce the risk of hardware failure.

In conclusion, this technique allowed obtaining a good correction of the deformity, brainstem decompression and CSF circulation at the CVJ in our patient. Despite the potential complications, this approach could treat BI and related problems in one single surgery and possibly prevent the need of shunting.

## Abbreviations

CSF: Cerebrospinal fluid
CVJ: Cranio-vertebral junction
CT: Computed tomography
OI: Osteogenesis imperfecta
BI: Basilar impression
MRI: Magnetic Resonance Imaging
